# How advanced is the epidemiological transition in Papua New Guinea? New evidence from verbal autopsy

**DOI:** 10.1093/ije/dyab088

**Published:** 2021-05-02

**Authors:** John D Hart, Viola Kwa, Paison Dakulala, Paulus Ripa, Dale Frank, Victor Golpak, Timothy Adair, Deirdre Mclaughlin, Ian D Riley, Alan D Lopez

**Affiliations:** 1 Melbourne School of Population and Global Health, University of Melbourne, Carlton, VIC, Australia; 2 National Department of Health, Islander Drive, Port Moresby, Papua New Guinea; 3 Western Highlands Provincial Health Authority, Mt Hagen, Papua New Guinea; 4 Milne Bay Provincial Health Authority, Alotau, Papua New Guinea; 5 West New Britain Provincial Health Authority, Kimbe, Papua New Guinea

**Keywords:** Verbal autopsy, mortality surveillance, cause of death, Papua New Guinea, global burden of disease

## Abstract

**Background:**

Reliable cause of death (COD) data are not available for the majority of deaths in Papua New Guinea (PNG), despite their critical policy value. Automated verbal autopsy (VA) methods, involving an interview and automated analysis to diagnose causes of community deaths, have recently been trialled in PNG. Here, we report VA results from three sites and highlight the utility of these methods to generate information about the leading CODs in the country.

**Methods:**

VA methods were introduced in one district in each of three provinces: Alotau in Milne Bay; Tambul-Nebilyer in Western Highlands; and Talasea in West New Britain. VA interviews were conducted using the Population Health Metrics Research Consortium (PHMRC) shortened questionnaire and analysed using the SmartVA automated diagnostic algorithm.

**Results:**

A total of 1655 VAs were collected between June 2018 and November 2019, 87.0% of which related to deaths at age 12 years and over. Our findings suggest a continuing high proportion of deaths due to infectious diseases (27.0%) and a lower proportion of deaths due to non-communicable diseases (NCDs) (50.8%) than estimated by the Global Burden of Disease Study (GBD) 2017: 16.5% infectious diseases and 70.5% NCDs. The proportion of injury deaths was also high compared with GBD: 22.5% versus 13.0%.

**Conclusions:**

Health policy in PNG needs to address a ‘triple burden’ of high infectious mortality, rising NCDs and a high fraction of deaths due to injuries. This study demonstrates the potential of automated VA methods to generate timely, reliable and policy-relevant data on COD patterns in hard-to-reach populations in PNG.

Key Messages

- The findings of this study validate the priorities of the health sector in tackling infectious, maternal and child mortality, as well as injuries and violence, and suggest that even greater effort would be beneficial in these areas, while also addressing the preventable causes of non-communicable diseases (NCDs).
- The Global Burden of Disease Study (GBD) appears to overestimate NCD mortality in Papua New Guinea (PNG).
- Verbal autopsy methods can generate policy-relevant cause of death data and should be routinely applied in PNG.

## Introduction

Timely and accurate cause of death (COD) data are essential to guide health policy debates and prioritize health investments, but this information is not available for the majority of deaths in Papua New Guinea (PNG). Over 80% of PNG’s population live in rural areas where access to health care is typically limited and few deaths are certified by health professionals. The proportion of deaths in the country that are recorded in health facilities has been estimated to be 26%; however, only a small fraction of these undergo medical certification of COD to produce useful mortality statistics.^1^ This results in incomplete mortality data, mostly from health facilities in urban areas, which are not representative of mortality conditions in the wider population.

PNG suffered epidemics of newly-introduced respiratory and diarrhoeal diseases in the late 19th and early 20th century, followed by mortality declines associated with increased access to health care and malaria vector control after the Second World War.^2^ Although there is uncertainty about the estimates for PNG, child mortality has steadily decreased from approximately 79.5 per 1000 live births in 1990 to 52.9 per 1000 live births in 2017; and life expectancy has increased over the same period from 57.3 to 61.2 in females and 52.0 to 56.2 in males.^3^ However, the extent to which the epidemiological transition has progressed, associated with decreasing infectious mortality in children and increased non-communicable diseases (NCDs) in adults, is unclear given the lack of data. This knowledge gap represents a serious challenge for informed health planning, with available studies suggesting there is likely to be significant variation in NCD prevalence across the Pacific region and significant differences in risk factors for NCDs within PNG.^4^^,^^5^

In this paper, we present data emanating from the application of automated verbal autopsy (VA) methods to help address the critical knowledge gap about COD patterns in PNG. This involves, first, a structured interview with relatives of the deceased regarding relevant and easily observable signs and symptoms experienced by the deceased before death. Second, automated diagnostic algorithms are now available to diagnose the COD based on artificial intelligence applied to pattern recognition, a development that has significantly reduced the time and cost, and improved comparability, of VA diagnoses compared with physician review.^6^

In close collaboration with the National Department of Health (NDOH) and Civil and Identity Registry (CIR), we have implemented innovative methods to improve mortality data, focusing particularly on improving the notification of community deaths in selected pilot sites and diagnosing their causes using automated VA methods. In this paper, we report findings from the application of VA in PNG, demonstrating that the method is feasible and can yield information of direct policy relevance.

## Methods

The work analysed in this study was conducted in collaboration with the Government of Papua New Guinea. As part of a government programme and posing no risk to participants, ethical approval was not required.

### Study sites

**Figure 1:**
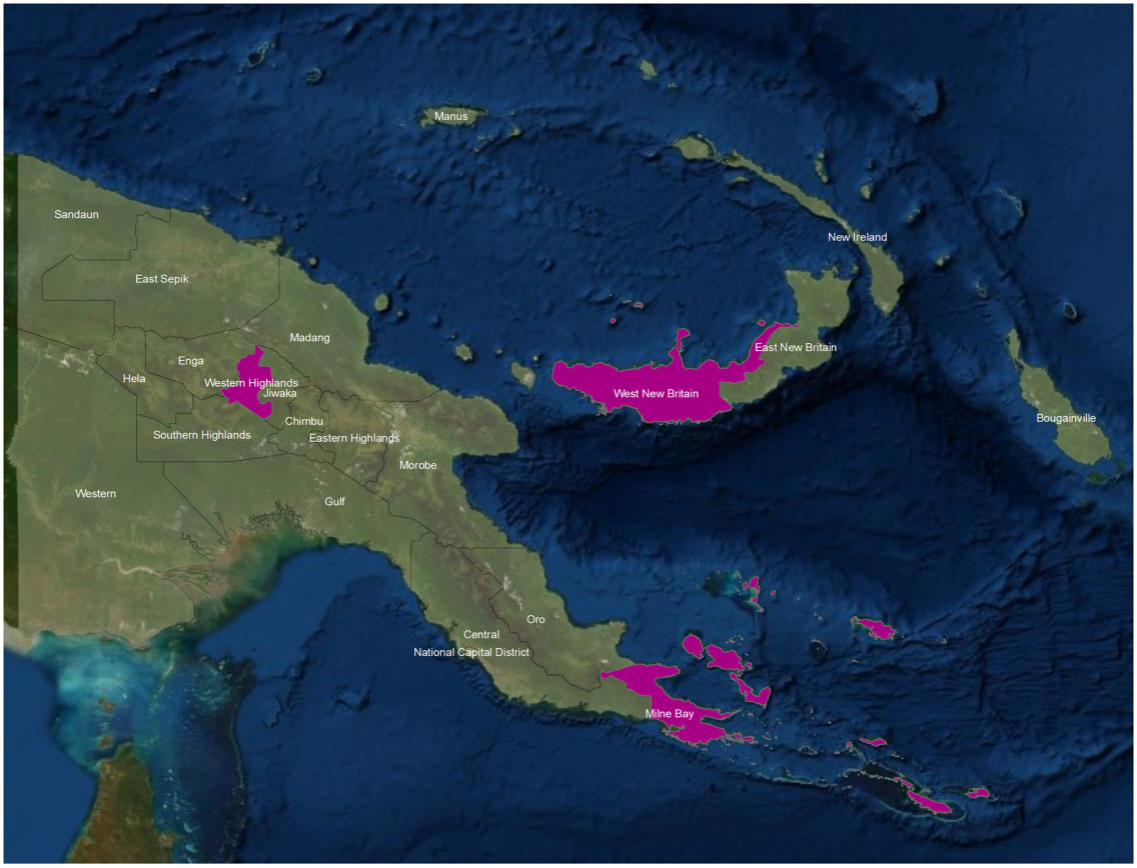
The provinces of Papua New Guinea which introduced verbal autopsy activities in 2018: Milne Bay, Western Highlands and West New Britain

VA was introduced in 2018 by Provincial Health Authorities (PHAs) in one district in each of three provinces in PNG: Alotau in Milne Bay; Tambul-Nebilyer in Western Highlands; and Talasea in West New Britain (see Figure 1). This was a purposive sample made, as far as possible, to represent PNG’s ecological, epidemiological and cultural diversity. However, the districts were not selected purely on the basis of representativeness, but also on whether there was sufficient local government support as well as experience with implementation of the electronic National Health Information System (eNHIS) which is planned as the vehicle for integration of VA data into a national mortality surveillance system.^7^ Random selection of sites was not attempted, as significantly more districts would be required in a country as diverse as PNG to ensure representative data.

Western Highlands is one of seven highlands provinces which lie at altitudes of 5000 feet and above and are home to 40% of the country’s population. West New Britain is one of five island provinces. Milne Bay lies at the eastern tip of mainland PNG. All three provinces are in the second quartile of provinces ranked by socioeconomic development indices.^8^ At the next level, selection of three districts in these provinces was based on their good communication with provincial headquarters. In short, the selective processes meant that the study would tend to overestimate rather than underestimate the extent of the epidemiological transition in PNG as a whole.

In some provinces, ward recorders (WRs) are engaged to document basic data from the villages in their ward, such as population movements, births and deaths. In Alotau and Talasea districts, we trained WRs to notify the nearest health centre of any community deaths that occurred in their ward, using either paper forms or mobile phones depending on the different capabilities of remote communities regarding transport, power and telecommunication connections. Deaths occurring in health facilities were excluded as these are recorded by the health system and should have a medical certificate of COD completed. Deaths were followed up by health workers trained in VA methodology. In Tambul-Nebilyer District there are no WRs, and hence village health volunteers (VHVs), involved in the delivery of newborn care, were recruited as death notification agents.

### Verbal autopsy tool

VA data were collected using the shortened Population Health Metrics Research Consortium (PHMRC) questionnaire.^9^ The questionnaire consists of age-specific modules and was administered using ODK software on Android devices. Each interview typically took about 20–25 min to complete.^10^ VA data were uploaded to an Aggregate server that was password-protected by NDOH.

### Data collection

VAs were conducted between June 2018 and November 2019. Three-day training courses for health workers on the VA methodology were facilitated by some of us (V.K., J.H.) and local VA master trainers. Training for WRs and VHVs as notifying agents was provided concurrently and lasted 1 day.

### Analysis

VA interviews were analysed using the SmartVA-Analyze program, which can identify 33 cause categories, as listed in Table 1.^11^ Cases where the response patterns to symptom questions were insufficiently clear for the algorithm to assign the most probable COD, (classified as ‘undetermined’) were redistributed to other VA causes based, equally, on: (i) the likelihood of each cause being assigned as undetermined in a gold-standard database of 12 542 deaths for which VA was performed and the true COD was known; and (ii) the estimated pattern of CODs for the country as reported in the Global Burden of Disease Study (GBD).^11^

**Table 1:** Adult causes of death as from SmartVA with corresponding International Statistical Classification of Diseases Tenth Revision (ICD-10) codes and cause of death categories for assessing the epidemiological transition

Disease category	Cause (ICD-10 code)
Endemic infections and	Malaria (B50-B54)
conditions of poverty	Diarrhoea/dysentery (A00-A09)
	Pneumonia (J10-J22, J85)
	Maternal (O00-O99)
	Other infectious diseases^>^
	All endemic infections
Emerging infections	TB (A15-19)
	AIDS (B20-B24)
	Cervical cancer (C53)
	All emerging infections
Endemic NCDs	Chronic respiratory disease (J40-J46)
	Leukaemia/lymphoma (C81-C85)
	Cirrhosis (K70-76)
	Chronic kidney disease (N17-N19)
	Breast cancer (C50)
	Stomach cancer (C16)
	Oesophageal cancer (C15)
	Colorectal cancer (C18-21)
	Prostate cancer (C61)
	Other NCDs^>^
	Other cancers^>^
	Other cardiovascular diseases^>^
	All endemic NCDs
Emerging NCDs	Diabetes (E10-E14)
	Stroke (I60-I69)
	Ischaemic heart disease (I20-I25)
	Lung cancer (C34)
	All emerging NCDs
Injuries	Homicide (X85-Y09)
	Falls (W00-W19)
	Road traffic (V01-V89)
	Drowning (W65-W74)
	Fires (X00-X19)
	Bite of venomous animal (X20-29)
	Poisonings (X40-49)
	Suicide (X60-84)
	Other injuries^>^

> ICD-10 codes for ‘Other’ disease categories available as Supplementary Table S1, available as Supplementary data at *IJE* online.

NCDs, non-communicable diseases.

We focus our analyses on deaths among adolescents and adults (12 years of age and older) due to low numbers of child deaths in the study. To provide some perspective on the plausibility of the results, we compare our findings with other COD analyses recently published for PNG, as well as GBD estimates.^1^^,^^12^^,^^13^ To do so, we have classified COD into five broad causes, following Gouda *et al.* (2019) (see Table 1). This categorization groups together emerging conditions that might be expected to increase in prominence with the epidemiological transition. Endemic infections and conditions of poverty (e.g. malaria and pneumonia) and emerging infections (e.g. TB and AIDS) correspond to GBD Group I; endemic NCDs (e.g. chronic respiratory disease and most cancers) and emerging NCDs (e.g. diabetes and ischaemic heart disease) correspond to GBD Group II; and injuries correspond to GBD Group III.

Expected deaths in each district were calculated using the estimated district populations (derived from the 2011 census and projected to the mid-point of the data collection period using the district’s population growth rate between the 2000 and 2011 censuses) and crude death rates (CDRs; deaths per 1000 population) derived from provincial mortality estimates (Milne Bay CDR = 7.2/1000; Western Highlands = 5.3/1000; West New Britain = 6.2/1000).^8^^,^^14^ The estimated population of Alotau District, Milne Bay, was 120 725; of Tambul-Nebilyer District, Western Highlands, 87 273; and of Talasea District, West New Britain, 246 585; totalling a little over 5% of the country’s estimated population of 8.8 million in 2019.^15^ Completeness was calculated as the number of VAs completed divided by the total expected deaths in each district for the period January 2018 to September 2019 (i.e. 21 months), as VAs were conducted retrospectively for any deaths during this period. The calculations tend to underestimate community death notification, as approximately one-quarter of deaths are expected to occur in health facilities and are thus ineligible for VA.^1^

## Results

A total of 1655 VAs were collected [87.0% adult and adolescent deaths (12 years of age and older), 7.7% child deaths (29 days to 11 years) and 5.3% neonatal deaths (0–28 days)], summarized by site in Table 2. The proportion of child deaths is lower than the GBD estimate of 11.9% and slightly lower for neonatal deaths (6.6%). This is expected since, in our experience in several countries, child deaths are disproportionately more likely than adult deaths to occur in health facilities, where we have not conducted VAs.

**Table 2:** Age distribution of verbal autopsies collected by site compared with Global Burden of Disease Study (GBD) country-level estimates of mortality, Papua New Guinea, 2018–19

Age category	Alotau	Talasea	Tambul-Nebilyer	Total	GBD
	*n* (%)	*n* (%)	*n* (%)	*n* (%)	estimate %
Adult and adolescent (12+ years)	478 (85.4)	471 (87.7)	491 (88.0)	1,440 (87.0)	81.5
Child (1 month—11 years)	42 (7.5)	39 (7.3)	47 (8.4)	128 (7.7)	11.9
Neonate (<28 days)	40 (7.1)	27 (5.0)	20 (3.6)	87 (5.3)	6.6
Total	560 (100)	537 (100)	558 (100)	1,655 (100)	100

Estimated completeness of death notification was 36.7% in Alotau, 20.2% in Talasea and 68.5% in Tambul-Nebilyer. COD was undetermined for 8.1% of the VAs, substantially lower than what is typically observed (about 15%) in other countries where SmartVA has been applied.^16^

The distribution of adult and adolescent mortality by site and broad cause categories is given in Table 3. Overall, these findings suggest a continuing high proportion of deaths due to infectious diseases (27.0%); a lower proportion of deaths due to NCDs (50.8%) than GBD estimates, and a comparatively high proportion of injury deaths (22.5%). By site, higher levels of emerging infections, particularly TB, were recorded for Talasea than for the other sites (14.7%). Tambul-Nebilyer in the Western Highlands recorded the highest endemic infections (22.4%), as well as the highest levels of emerging NCDs (25.9%) and lowest emerging infections (4.7%). Injury deaths were highest in Tambul-Nebilyer (35.5%) and lowest in Talasea (13.2%).

**Table 3:** Cause-specific mortality fractions by five broad cause categories and site, Papua New Guinea, 2018–19

Cause classification	Alotau	Talasea	Tambul-Nebilyer	Total
	%	%	%	%
Endemic infections	18.1	14.1	22.4	18.4
Emerging infections	6.4	14.7	4.7	8.6
All infections	24.5	28.8	27.1	27.0
Endemic NCDs	39.4	39.5	11.4	30.1
Emerging NCDs	17.3	18.4	25.9	20.7
All NCDs	56.7	57.9	37.3	50.8
All Injuries	19.0	13.2	35.5	22.5
Total	100.0	100.0	100.0	100.0

NCDs, non-communicable diseases.

The distribution of deaths by age and sex across the five broad cause categories is shown in Table 4. Infectious mortality was lower in older females: 20.5% in the 65+ years age group compared with 43.5% in those aged 12–44 years. Mortality from endemic infections and conditions of poverty was higher in females (24.0%) than males (14.9%); the major contributor to the difference was maternal causes, comprising 4.1% of all female deaths (and 10.0% of female deaths at ages 12–44). The proportion of deaths due to NCDs increased with age, accounting for 30.6% of male deaths and 33.0% of female deaths at ages 12–44 years, and twice that (66.0% for males and 68.1% for females) at ages 65 years and over. Endemic and emerging NCD mortality did not differ greatly by sex. Injury deaths were considerably higher at younger ages, particularly for males, accounting for 45.2% of male deaths at ages 12–44 years but only one in 10 (10.1%) at ages 65 years and over.

**Table 4:** Cause-specific mortality fractions in adolescents and adults (12 years of age and older) for five broad cause categories by age and sex, Papua New Guinea, 2018–19

Cause classification	Male (%)				Female (%)			
	12-44 years	45-64 years	65+ years	All 12+	12-44 years	45-64 years	65+ years	All 12+
Endemic infections	16.8	13.1	14.5	14.9	30.4	25.6	15.0	24.0
Emerging infections	7.6	8.7	9.0	8.4	13.1	7.8	5.5	8.7
All infections	24.4	21.8	23.5	23.3	43.5	33.4	20.5	32.7
Endemic NCDs	18.4	30.7	38.6	28.8	19.3	34.1	42.0	31.3
Emerging NCDs	12.2	23.1	27.4	20.8	13.7	22.1	26.1	20.1
All NCDs	30.6	53.8	66.0	49.6	33.0	56.2	68.1	51.4
All injuries	45.2	24.3	10.1	27.0	23.7	10.3	11.3	15.8
Total	100.0	100.0	100.0	100.0	100.0	100.0	100.0	100.0

NCDs, non-communicable diseases.

More detail on the distribution of specific CODs for adults and adolescents is given in Table 5. For males, the five leading CODs were, in descending order: chronic respiratory disease, stroke, ischaemic heart disease (IHD), malaria and a residual category of ‘other NCDs’ (other than those included in the VA cause list); and for females: chronic respiratory disease, malaria, pneumonia, IHD and stroke. For both sexes, the leading causes of adult death in PNG were a mixture of chronic and infectious diseases. Although not in the top five causes, several specific causes of injury deaths also figured prominently, including road traffic accidents, poisoning, homicide and drowning.

**Table 5:** Cause-specific mortality fractions in adolescents and adults (12 years of age and older) by broad age group, and Global Burden of Disease Study (GBD) estimates, Papua New Guinea, 2018–19

Cause category	Cause (ICD-10 code)	12--4 years				45+ years				Total VAs (12+ years)				GBD (2017)	
		Male		Female		Male		Female		Male		Female		Male	Female
				
		*n* ^a^	%^b^	*n* ^a^	%^b^	*n* ^a^	%^b^	*n* ^a^	%^b^	*n* ^a^	%^b^	*n* ^a^	%^b^	%	%
Endemic infections	Malaria (B50-B54)	31	10.6	16	8.7	23	4.6	25	7.9	54	6.6	42	8.3	1.0	0.4
	Diarrhoea/dysentery (A00-A09)	2	0.9	0	0.4	7	1.7	11	4.1	9	1.4	11	2.7	2.2	3.3
	Pneumonia (J10-J22, J85)	12	4.2	17	9.0	35	6.6	21	6.8	48	5.9	39	7.7	6.1	4.6
	Maternal (O00-O99)	0	0.0	19	10.0	0	0.0	3	0.9	0	0.0	22	4.1	0.0	5.5
	Other infectious diseases^c^	3	1.1	4	2.3	4	0.9	0	0.3	7	1.0	5	1.2	1.4	1.6
	All endemic infections	48	16.8	56	30.4	69	13.8	60	20.0	118	14.9	119	24.0	9.7	15.0
Emerging infections	TB (A15-19)	16	5.4	8	4.2	45	8.0	7	2.2	62	7.1	15	2.9	1.6	1.5
	AIDS (B20-B24)	6	2.2	11	5.8	4	0.9	2	0.8	10	1.3	13	2.5	1.3	2.3
	Cervical cancer (C53)	0	0.0	6	3.1	0	0.0	12	3.5	0	0.0	18	3.3	0.0	2.1
	All emerging infections	22	7.6	25	13.1	49	8.9	21	6.5	72	8.4	46	8.7	2.9	5.9
Endemic NCDs	Chronic respiratory disease (J40-J46)	9	3.2	8	4.4	100	18.4	58	17.8	111	13.2	67	13.0	17.5	16.2
	Leukaemia/lymphoma (C81-C85)	2	0.7	3	1.7	3	0.7	4	1.4	5	0.7	8	1.6	0.4	0.5
	Cirrhosis (K70-76)	10	3.4	2	1.2	12	2.3	4	1.4	22	2.7	6	1.3	2.3	1.1
	Chronic kidney disease (N17-N19)	8	2.8	1	0.7	13	2.6	7	2.5	21	2.6	8	1.8	2.5	3.0
	Breast cancer (C50)	0	0.0	9	4.7	0	0.0	13	3.8	0	0.0	22	4.1	0.0	1.9
	Stomach cancer (C16)	1	0.4	2	1.1	0	0.1	0	0.1	1	0.2	2	0.4	1.1	0.9
	Oesophageal cancer (C15)	1	0.4	2	1.1	12	2.1	8	2.4	13	1.5	10	1.9	0.1	0.1
	Colorectal cancer (C18-21)	1	0.4	0	0.1	1	0.4	2	0.9	2	0.4	2	0.6	0.5	0.6
	Prostate cancer (C61)	0	0.0	0	0.0	6	1.2	0	0.0	6	0.8	0	0.0	0.5	0.0
	Other NCDs^c^	20	6.9	7	4.0	31	6.0	23	7.4	51	6.2	30	6.0	3.3	4.0
	Other cancers^c^	0	0.1	0	0.1	1	0.4	0	0.3	1	0.3	0	0.2	2.7	3.6
	Other cardiovascular diseases^c^	0	0.1	0	0.2	0	0.3	0	0.4	0	0.2	0	0.4	4.9	7.8
	All endemic NCDs	52	18.4	34	19.3	179	34.5	119	38.4	233	28.8	155	31.3	35.8	39.7
Emerging NCDs	Diabetes (E10-E14)	2	0.8	4	2.2	33	6.1	23	7.3	35	4.2	27	5.4	3.3	5.9
	Stroke (I60-I69)	15	5.2	11	6.0	61	11.3	23	7.6	78	9.3	34	6.9	10.9	11.7
	Ischaemic heart disease (I20-I25)	18	6.2	10	5.4	39	7.5	28	8.9	58	7.1	38	7.5	17.4	13.2
	Lung cancer (C34)	0	0.0	0	0.1	1	0.3	1	0.4	1	0.2	1	0.3	2.1	0.9
	All emerging NCDs	35	12.2	25	13.7	134	25.2	75	24.2	172	20.8	100	20.1	33.7	31.7
Injuries	Homicide (X85-Y09)	17	5.8	6	3.2	14	2.6	5	1.6	31	3.6	12	2.3	1.8	0.6
	Falls (W00-W19)	7	2.4	0	0.1	16	2.9	6	1.9	23	2.7	6	1.2	0.5	0.2
	Road traffic (V01-V89)	33	11.1	7	3.8	18	3.3	2	0.7	51	5.9	10	2.0	5.4	2.7
	Drowning (W65-W74)	24	8.1	6	3.2	7	1.4	0	0.2	34	4.0	6	1.2	2.3	0.6
	Fires (X00-X19)	4	1.4	3	1.6	3	0.6	1	0.4	7	0.9	4	0.8	0.2	0.3
	Bite of venomous animal (X20-29)	5	1.7	0	0.0	1	0.2	0	0.0	6	0.7	0	0.0	0.4	0.2
	Poisonings (X40-49)	19	6.4	9	4.8	14	2.7	4	1.5	33	3.9	13	2.6	0.2	0.1
	Suicide (X60-84)	6	2.1	5	2.7	5	8.0	4	1.2	11	1.3	10	1.9	3.7	1.6
	Other injuries^c^	18	6.2	8	4.3	15	2.9	10	3.3	33	4.0	19	3.8	2.3	1.1
	All injuries	133	45.2	44	23.7	93	17.5	32	10.8	229	27.0	80	15.8	16.8	7.4
	Undetermined	11	–	11	–	53	–	41	–	64	–	52	–	–	–
Total		301	100	195	100	577	100	348	100	888	100	552	100	100	100

NCDs, non-communicable diseases.

ICD-10, International Statistical Classification of Diseases Tenth Revision

a Absolute numbers of verbal autopsies before redistribution of undetermined cases (note age was unknown for 19 adult cases).

b Cause-specific mortality fractions following redistribution of undetermined cases.

c ICD-10 codes for ‘Other’ disease categories available as Supplementary Table S1, available as Supplementary data at *IJE* online.

A comparison of the community mortality patterns with recently published VA research in PNG is shown in Table 6. The COD distribution is similar between the three VA collection sites in this study and the four, quite different but smaller, sites used in the Gouda *et al.* (2019) study: approximately one-quarter of adult mortality due to infection and half due to NCDs. These findings are in stark contrast to the GBD, which estimates only 16.5% of adult deaths were due to infections, with seven in 10 (70.5%) due to NCDs. Further, injury-related deaths accounted for 22.5% and 22.3% of adult deaths in this study and the Gouda *et al*. data, respectively, compared with only 13.0% from the GBD estimates.

**Table 6:** Cause-specific mortality fractions by disease category in adolescents and adults (12 years of age and older) comparing mortality surveillance sites in this study with those reported by Gouda *et al*., with redistribution of undetermined cases, and Global Burden of Disease Study (GBD) estimates, Papua New Guinea

Cause classification	Data source (year of estimate)									
	This study (2018-19)				Verbal autopsy data from Gouda *et al*. 2019 (2009-14)					GBD (2017)
	Alotau (*n = *478)	Talasea (*n *= 471)	Tambul-Nebilyer (*n *= 491)	Total (*n *= 1,440)	West Hiri (*n* = 221)	Asaro (*n* = 332)	Karkar (*n* = 226)	Hides (*n* = 89)	Total (*n *= 868)	
	**%**	**%**	**%**	**%**	**%**	**%**	**%**	**%**	**%**	**%**

Endemic infections	18.1	14.1	22.4	18.4	12.3	15.0	12.1	16.1	12.8	12.4
Emerging infections	6.4	14.7	4.7	8.6	17.3	11.5	11.2	7.9	12.5	4.1
All infections	24.5	28.8	27.1	27.0	29.6	26.5	23.3	24.0	25.3	16.5
Endemic NCDs	39.4	39.5	11.4	30.1	22.7	33.7	36.4	42.5	32.5	37.4
Emerging NCDs	17.3	18.4	25.9	20.7	22.1	18.8	18.2	9.8	20.0	33.1
All NCDs	56.7	57.9	37.3	50.8	44.8	52.5	54.6	52.3	52.5	70.5
All injuries	19.0	13.2	35.5	22.5	25.6	20.8	21.9	23.7	22.3	13.0
Total	100.0	100.0	100.0	100.0	100.0	100.0	100.0	100.0	100.0	100.0

NCDs, non-communicable diseases.

COD estimates by site are shown in Table 7. These data are presented to aid our understanding of the broad cause patterns and inform our discussion of the epidemiological transition in PNG. They should be viewed with caution given the relatively small sample sizes (approximately 500 deaths per site).

**Table 7:** Causes of death by site in adolescents and adults (12 years of age and older), Papua New Guinea, 2018–2019

Cause	Alotau (Milne Bay)		Talasea (West New Britain)		Tambul-Nebilyer (Western Highlands)		Total	
	*n* ^a^	%^b^	*n* ^a^	%^b^	*n* ^a^	%^b^	*n* ^a^	%^b^
Chronic respiratory	96	21.4	57	13.1	25	5.1	178	13.1
Stroke	15	4.2	19	4.8	78	15.9	112	8.4
Ischaemic heart disease	29	7.1	19	4.9	48	9.8	96	7.3
Malaria	19	4.8	12	3.3	65	13.3	96	7.2
Pneumonia	31	7.3	20	4.9	36	7.3	87	6.6
Other NCDs	23	5.7	57	12.8	1	0.2	81	6.2
TB	18	4.0	50	10.8	9	1.8	77	5.5
Diabetes	23	5.4	38	8.5	1	0.2	62	4.7
Road traffic	1	0.4	16	3.6	44	9.0	61	4.4
Other injuries	15	3.6	5	1.5	32	6.5	52	3.9
Poisonings	1	0.6	5	1.4	40	8.2	46	3.4
Homicide	14	3.2	13	3.0	16	3.3	43	3.1
Drowning	12	2.7	8	1.9	20	4.1	40	2.9
Chronic kidney disease	3	1.1	12	3.0	14	2.9	29	2.3
Cirrhosis	14	3.3	11	2.6	3	0.6	28	2.2
Falls	21	4.6	5	1.2	3	0.6	29	2.1
Diarrhoea/dysentery	8	2.6	12	3.2	0	0.0	20	1.9
AIDS	5	1.3	4	1.1	14	2.9	23	1.8
Oesophageal cancer	9	2.0	14	3.1	0	0.0	23	1.7
Breast cancer	3	0.7	9	2.0	10	2.0	22	1.6
Maternal	11	2.4	8	1.8	3	0.6	22	1.6
Suicide	9	2.0	1	0.3	11	2.2	21	1.5
Cervical cancer	5	1.1	13	2.8	0	0.0	18	1.3
Leukaemia/lymphomas	6	1.5	4	1.1	3	0.6	13	1.1
Other infectious diseases	3	1.0	3	0.9	6	1.2	12	1.1
Fires	4	1.0	0	0.1	7	1.4	11	0.8
Colorectal cancer	4	1.2	0	0.3	0	0.0	4	0.5
Prostate cancer	5	1.2	1	0.3	0	0.0	6	0.5
Bite of venomous animal	4	0.9	1	0.2	1	0.2	6	0.4
Lung cancer	2	0.6	0	0.2	0	0.0	2	0.3
Other cancers	0	0.3	1	0.5	0	0.0	1	0.3
Other cardiovascular diseases	0	0.5	0	0.4	0	0.0	0	0.3
Stomach cancer	2	0.5	1	0.3	0	0.0	3	0.3
Undetermined	63	–	52	–	1	–	116	–
Total	478	100	471	100	491	100	1440	100

a Absolute numbers of verbal autopsies before redistribution of undetermined cases.

b Cause-specific mortality fractions following redistribution of undetermined cases.

## Discussion

This paper reports, for the first time, VA data collected in PNG through official channels in collaboration with NDOH, the PHAs and CIR. A total of 1655 VAs were collected from Alotau, Talasea and Tambul-Nebilyer districts, representing three of the four regions of PNG—Southern, Islands, and Highlands Regions—between June 2018 and November 2019. Importantly, our results suggest that the epidemiological transition in PNG is likely to be far less advanced than other estimates, such as the GBD, suggest.

The VA data indicated higher infectious and injury-related mortality, and lower NCD mortality, than the GBD 2017 estimates.^13^ Endemic NCD mortality (30.1%) was slightly lower than the GBD estimate (37.4%), whereas mortality from emerging NCDs (20.7%) was considerably lower (33.1%), reflecting the earlier stage of the epidemiological transition in PNG measured directly from community deaths than predicted by the GBD models. Our data confirm the dual burden of infectious and NCD mortality now facing health policy makers in PNG, as recently proposed by Kitur *et al*.^1^ Arguably, policy makers in PNG are now faced with a ‘triple’ burden, given the extraordinarily high fraction of adult and adolescent deaths (about one in four) that are due to injuries, far higher than is generally observed in countries at this early stage of epidemiological transition and most of which occur in younger adults. In contrast, the GBD models that indicate waning prevalence of infectious diseases, and relatively low injury mortality, might suggest significantly greater policy emphasis be given to controlling NCDs.

Chronic respiratory disease and stroke mortality were both lower than the GBD estimates, and IHD mortality was less than half what the GBD estimated, accounting for much of the difference between the VA and GBD estimates of emerging NCDs. Diabetes mortality was similar to GBD estimates overall, although it varied considerably by site, being highest in Talasea and lowest in Tambul-Nebilyer. Chronic respiratory disease and stroke also varied by site, with a predominance of chronic respiratory disease in Alotau and of stroke in Tambul-Nebilyer. NCD risk factors vary considerably in places such as Alotau, where much of the population is only reachable by air or sea, compared with peri-urban populations.^4^ Interestingly, IHD and stroke mortality were relatively high in the 12–44 year age group and did not increase greatly in those aged 45+, perhaps reflecting increased NCD risk factors and limited access to preventive interventions, leading to the development of cardiovascular disease at relatively young ages.^17^

The higher infectious mortality identified from VA in this study, compared with that estimated by the GBD models, was largely driven by pneumonia, malaria, TB and HIV mortality. Malaria mortality varied considerably by site, as would be expected, and was highest in Tambul-Nebilyer. Malaria transmission is unstable in Western Highlands Province, and the population suffer epidemics unlike the other VA collection sites where transmission is stable and adults generally have immunity. The latest malaria indicator survey in PNG reported a 9-fold increase in the prevalence of infection to 7.1% in the at-risk population, between 2014 and 2017, supporting our finding that malaria remains a major concern.^18^ HIV infection and mortality are believed to be generally under-reported in PNG due to stigma experienced by the individual and family. A recent study has indicated that prevalence of HIV in at-risk populations may be as high as 20%, with the majority undiagnosed at the time of the survey.^19^ There were considerable variations in HIV and TB mortality by site in this study, with higher HIV in Tambul-Nebilyer and higher TB in Talasea, consistent with local surveillance data suggesting steady increases in TB since 2008.^20^ Activities such as palm oil production with high levels of migrant labour in Talasea, and the trading economy along the Highlands Highway connecting the Highlands Region and northern coastal cities, are likely to contribute to these patterns.

Injury-related mortality was nearly two times higher than the GBD estimates and specific causes differed by site, with notably higher road traffic and poisoning deaths in Tambul-Nebilyer. There is a more extensive road network in Tambul-Nebilyer than the other sites and several recent major accidents have occurred. Homicide deaths were relatively high in all sites, accounting for 3.1% of adult deaths overall, and were due to piracy, tribal conflicts and payback related to accusations of sorcery. Poisoning deaths may be more readily reported by respondents than elsewhere due to traditional beliefs in sorcery or witchcraft. Drowning deaths were also relatively high at all sites, 2.9% overall, occurring in wells and fast-flowing rivers in inland areas, as much as at sea. A more reliable understanding of mortality due to suicide, which accounted for 1.5% of all deaths, may be an important outcome from VA as these deaths are usually under-reported to hospitals due to both the immediacy of the death and stigma.

Despite relatively low numbers of VAs, the site-specific data suggest that different surveillance sites may be at different stages of the epidemiological transition. This is likely to be influenced by the huge socioeconomic, demographic and cultural heterogeneity that exists between and even within districts in PNG.^21^ The high endemic infections alongside high emerging NCDs in Tambul-Nebilyer are an example of how PNG’s diversity and multiple local factors may influence progression of the epidemiological transition. The policy value of VA, as it is implemented in additional provinces, may lie as much in understanding that granularity, as improving country-level COD estimates.

Our findings are broadly in agreement with the COD patterns from the smaller VA study reported by Gouda *et al.* (2019), using similar VA techniques (reproduced in Table 6). This provides confidence in the reproducibility of the SmartVA methodology that is currently being adopted more widely in PNG.^12^ In addition, Kitur *et al*. recently published COD patterns that include child and neonatal deaths, as well as both hospital and community deaths (as reported in Gouda *et al*.).^1^ They report higher infectious mortality (41–49%), lower NCD mortality (42–45%) and lower injury-related mortality (8–13%) than the VA data, which is expected given the inclusion of child deaths and the fact that fewer acute deaths from external causes are reported to health facilities.

The completeness of community death notification, as a percentage of all deaths, ranged from 20.2% to 68.5% in the pilot districts. Prior to the intervention, notification of community deaths was rare, occurring only if required for insurance or inheritance purposes. The variation in completeness in this study reflects the massive logistical challenges faced regarding data collection in remote areas of PNG. Every effort was made to facilitate death notification from all areas of each surveillance district, the strategies for which have been described in detail elsewhere.^22^ However, potential biases remain. As is common for reporting of deaths, including in VA programmes, just over 60% of the VAs in this study were conducted for male deaths, revealing a possible bias against female deaths.^16^ It is also feasible that deaths which were well known to a community, such as due to road traffic accidents, might have been more commonly reported, and that illnesses with stigma associated, such as related to chronic wasting, might have been under-reported. Further improving completeness of death notification is essential for reducing bias in COD data, and local government leadership and identification of appropriate notification agents will be central to improvements in completeness of death notification.^23^

Our findings need to be interpreted with caution given the limitations of the study. Reliable medical certification of COD is the gold standard for determining COD. However, an interim alternative to meeting the information needs of policy makers might well involve VA methods; SmartVA can reliably differentiate 33 adult, 22 child and six neonatal CODs. Although less specific than medical certification, the CODs identified by SmartVA represent the majority of important causes for health planning and policy. Due to relatively low numbers of VAs, the main focus of this study was on broad cause categories, and assessed individual CODs for the three implementation sites combined, to reduce random variation in the estimates. For some diseases, including cancers, with mortality fraction estimates between 0% and 2%, the number of VAs was too low to produce reliable estimates.

A potential shortcoming of this study is that we did not attempt to analyse the VA data using other publicly available diagnostic algorithms, such as InterVA and InSilicoVA. SmartVA, which analyses data from the shortened PHMRC questionnaire, was adopted by the Provincial Health Authorities in PNG for several reasons. First, the questionnaire contains approximately 50% fewer questions than the World Health Organization 2016 questionnaire that is required to run InterVA and InSilicoVA, which may represent an important time saving when introduced into routine health worker activities.^9^ Second, SmartVA had been used in PNG previously and the method is also used by the PNG Institute of Medical Research for its research, so for future comparisons, and consistency with current VA research applications, it was thought preferable to use the same instrument.^12^ The use of the PHMRC questionnaire does not facilitate analysis by InterVA and InSilicoVA. However, there is no consensus as to which algorithm may be most accurate and, indeed, if the data were analysed using three algorithms, resulting in different diagnoses for a significant fraction of cases, this would pose additional challenges for interpretation by the government in PNG.^6^^,^^24^

In addition to the Gouda *et al.* VA data, GBD estimates were used as an additional comparator. The comparison with GBD was not conducted to try and replicate the results, but rather because the GBD estimates are the only external source of information on COD patterns in the country. GBD estimates are derived from models of disease and covariate relationships and ‘borrow strength’ from existing regional data sources when estimating COD patterns for a particular country.^25^ In the case of PNG, this implies basing the estimates on vital statistics from Fiji, Samoa and Tonga, all of which have established NCD epidemics. In PNG, the relationship between covariates and mortality patterns which is the basis of the GBD COD modelling approach may be less valid than elsewhere, perhaps also related to the heterogeneous socio-cultural, economic and epidemiological diversity that characterizes the country and which is more difficult for disease models to capture.

The VA data reported here demonstrate the plausibility and feasibility of VA methods to generate timely, policy-relevant data on changing COD patterns in PNG. The greatest benefit from wider implementation of VA may relate to improved understanding of the variation between sites, enabling provinces to tailor health policy to their specific requirements. Important progress has been made towards incorporating VA methods into routine mortality surveillance activities, with the government endorsing VA as the method to be used for collecting cause of death data for community deaths in future. As eNHIS is rolled out in PNG, the inclusion of SmartVA, which has been shown to work well in PNG, along with sustainable and efficient methods for vital event notification, will facilitate the continuous flow of information on who dies of what, at what age, which is fundamental to guide the efficient allocation of resources to support health development across the country.

## Supplementary Data

Supplementary data are available at *IJE* online.

## Supplementary Material

dyab088_Supplementary_Data

## Data Availability

The data underlying this study belong to the Government of Papua New Guinea, who should be contacted directly for access to the data.
