# Supplementary material for: How advanced is the epidemiological transition in Papua New Guinea? New evidence from verbal autopsy
Source: Int J Epidemiol. 2021 May 2;50(6):2058–69. doi: 10.1093/ije/dyab088 (PMC8743130; doi:10.1093/ije/dyab088)
Supplement: dyab088_Supplementary_Data [file dyab088_supplementary_data.docx]

Supplementary table S1: International Statistical Classification of Diseases Tenth Revision (ICD-10) codes for the SmartVA ‘Other’ disease categories

| **Cause of Death** | **ICD-10 Code** |
| --- | --- |
| Other Infectious Diseases | A10-A14, A20-B19, B25-B49, B55-B99 |
| Other Cancers | C00-C14, C17, C22-C33, C35-C49, C51-C52, C54-C60, C62-C80, C86-C90, C97-D48 |
| Other Cardiovascular Diseases | I00-I19 I26-I59, I70-I99 |
| Other Injuries | S00-T98, V90-V99, W20-W64, W75-W99, X30-X39, X50-X59, Y10-Y98 |
| Other Non-Communicable Diseases | All other ICD-10 codes if greater than 12 years |
